# Effects of Ce-Rich Mischmetal on Microstructure Evolution and Mechanical Properties of 5182 Aluminum Alloy

**DOI:** 10.3390/ma12244230

**Published:** 2019-12-17

**Authors:** Tianhao Gong, Junhui Dong, Zhiming Shi, Xinba Yaer, Huimin Liu

**Affiliations:** 1 School of Materials Science and Engineering, Inner Mongolia University of Technology, Hohhot 010051, China; 2 Inner Mongolia Key Laboratory of Light Metal Materials, Hohhot 010051, China

**Keywords:** 5182 Al Alloy, Ce-rich mischmetal, microstructure evolution, strengthening effect, mechanical property

## Abstract

This paper addresses the effects of Ce-rich mischmetal on the microstructure evolution of a 5182 aluminum alloy during annealing and rolling processes. The Ce-rich mischmetal was added to an as-cast 5182 aluminum alloy in an induction furnace, and this was followed by homogenized annealing at 450 °C for 24 h and a rolling operation. The microstructure evolution and mechanical properties’ analysis of the 5182 Al alloy were characterized. The results show that the Ce-rich mischmetal could modify the microstructure, refine the α-Al grains, break the network distribution of Mg_2_Si phases, and prevent Cr and Si atoms from diffusing into the Al_6_(Mn, Fe) phase in the as-cast 5182 Al alloys. Ce-rich mischmetal elements were also found to refine the Al_6_(Mn, Fe) phase after cold rolling. Then, the refined Al_6_(Mn, Fe) particles inhibited the growth of recrystallization grains to refine them from 10.01 to 7.18 μm after cold rolling. Consequently, the tensile strength of the cold-rolled 5182 Al alloy increased from 414.65 to 454.34 MPa through cell-size strengthening, dislocation density strengthening, and particle strengthening. The tensile strength of the recrystallization annealed 5182 Al alloy was increased from 322.16 to 342.73 MPa through grain refinement strengthening, and this alloy was more stable after the recrystallization annealing temperature.

## 1. Introduction

5182 aluminum alloys have been commonly used to fabricate car bodies and complex shaped parts on vehicles for the automotive industry due to their high strength-to-weight ratio, light weight, weldability, and corrosion resistance properties [1]. However, the increasingly strict standards of lightweight automotive technology highly require the mechanical strength of alloys [2]. A number of investigations have achieved the goal of improving alloy strength in the as-cast 5182 aluminum alloy through the microalloying [3,4,5,6,7,8,9,10,11,12] and laser sintering methods [13,14].

Several researchers [5,6,7,8,9,10,11,12] have investigated the effect of Al–5Ti–1B, Zr, and Sc on the microstructure and tensile strengths of Al alloys. Studies have shown that more nucleation sites are provided by intermetallic such as Al_3_Ti, Al_3_Zr, and Al_3_Sc. The grains are refined during solidification, while the tensile strength (UTS) and elongation (EI) values of the cast alloy are significantly improved after solidification. Daniele et al. [13,14] investigated the effects of particle size on the mechanical properties of sintered layers. Their study showed that optimum strength was associated on the largest neck size. Wang et al. [15] investigated the effects of Zn on the structure and tensile properties of an Al–Mg–Si–Cu alloy. Their study showed that adding Zn could influence the precipitate distribution and improve the UTS and EL values of the cast alloy. Du et al. [3] studied the influence of Ce addition on the microstructure and properties of a Al-Cu-Mn-Mg-Fe lithium battery shell alloy and found that Al_6_(Mn, Fe) precipitates could be remarkably refined by adding Ce. Medvedev et al. [16] studied the effect of La on the microstructure and mechanical properties of the 6xxx series type aluminum alloy and found that lanthanum inhibited the formation of the AlFeSi phase and reduced the grain size in as-cast alloys.

The addition of modifiers could reduce the grain size of or refine intermetallic compounds; among them, Ce not only affects the grain size but also affects the morphology and distribution of the second phase in the Al alloy [1,7]. Previous studies of our group have shown that Ce-rich mischmetal can refine as-cast 5182 Al alloy grains and improve their secondary phase morphology [17,18]. However, to our best knowledge, the microstructure and properties evolution after the cold deformation process of the Ce-rich mischmetal-modified 5182 aluminum alloy has rarely been studied. It is therefore of interest to study the effect of Ce-rich mischmetal on the microstructure and properties in the follow-up process. In this study, the effect of Ce-rich mischmetal on the microstructure evolution of the as-cast, homogenized annealed and cold-rolled 5182 Al alloys were characterized, and then their mechanical properties after the recrystallization and cold rolling process were systemically investigated.

## 2. Materials and Methods

The material used in the present study was a commercial 5182 Al alloy, which was supplied by Baotou Aluminum Co. Ltd. (Baotou, China) Ce-rich mischmetal was added as the master alloy to the Al alloy. Table 1 shows the composition of Ce-rich mischmetal.

**Figure 1:**
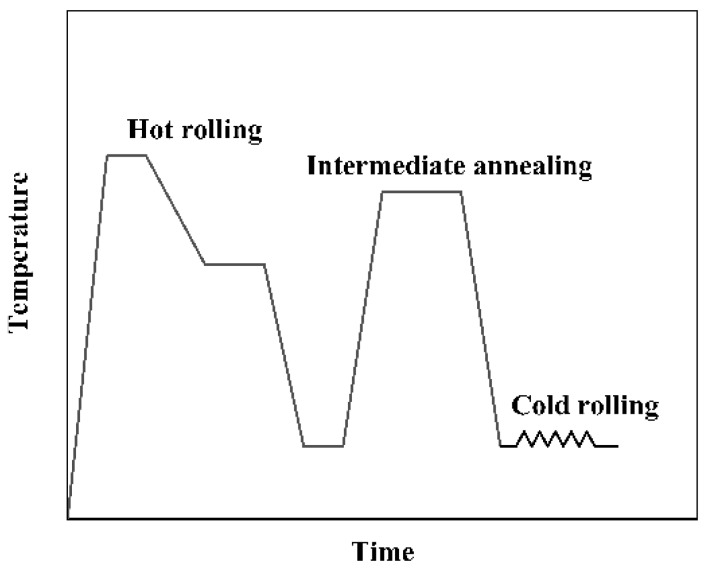
Flowchart for the rolling process of the 5182 Al alloy.

First, the 5182 Al alloy was melted in the induction furnace, where the melting temperature was 720 °C. After the Al alloy was entirely melted, the Ce-rich master alloy was added, and Mg was compensated for according to its burning loss during the melting process. After standing for 10 min, when the alloy elements in the melt were entirely dissolved, it was cast into Al plates with the dimensions of 35 × 270 × 320 mm (the compositions are shown in Table 1 and Table 2). After homogenized annealing at 450 °C for 24 h, the material surface was milled, and the final dimensions were 30 × 250 × 300 mm. The process for rolling of the Al alloy plate is shown in Figure 1. The starting temperature of the hot rolling was 500 °C, and the end temperature was 300 °C. The Al alloy plate was rolled from 30 to 3 mm after 20 passes. After annealing at 450 °C for one hour, the Al plate was cold-rolled down to 1 mm. In this paper, the unmodified 5182 Al alloy is designated as 5182-0Ce, while the modified 5182 Al alloy is designated as 5182-0.4Ce.

To study the recrystallization behavior of the Al alloy, the cold-rolled Al alloy plates were annealed at 330, 340, 350, 370, 380, 390, and 400 °C for 1 h. The standard tensile test samples were cut along the rolling direction. The mechanical properties (tensile strength and ductility) were determined by a universal testing machine (5565, Instron, Boston, MA, America) under a loading velocity of 0.2 mm/min and at room temperature. The final value of the tensile strength and ductility of each test was the average of six samples.

The microstructure was observed under an optical microscope (OM, DM4p, Leica, Frankfurt, Germany) fitted with a camera that used polarized and unpolarized light. The distribution of particles in the Al matrix was investigated by SEM (S-3400, Hitachi, Tokyo, Japan) coupled with energy-dispersive X-ray diffraction (EDS), and TEM (Tecnai G2 F20, FEI, Hillsboro, America) coupled with energy-dispersive X-ray diffraction (EDS). The TEM samples were polished down to 0.1 mm and then ion milled. An SEM (Quanta 650 FEG, FEI, Hillsboro, America), along with a field emission gun equipped with the Nordlys Nano Electron Backscattered Diffraction (EBSD) system, was used to observe the α-Al phase. The samples were electrolyzed for 90 s at −20 °C (cooled using liquid N_2_) at a voltage of 30 V in an electrolyte bath containing 15 mL of HClO_4_ and 285 mL of C_2_H_6_O. Channel 5 software was employed for data analysis [17].

## 3. Results

### 3.1. Mechanical Properties

**Figure 2:**
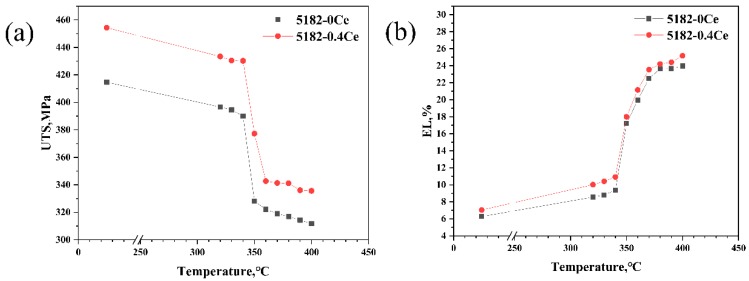
Relationship between the mechanical properties and annealing temperature for the 5182 Al alloys before and after Ce-modification: (**a**) tensile strength and (**b**) elongation.

Figure 2 shows the variations in the mechanical properties of the 5182 Al alloy before and after modification. After being annealed at different temperatures, the UTS and EL values of the 5182-0.4Ce Al alloy were higher than the 5182-0Ce Al alloy. The UTS of the 5182-0.4Ce Al alloy after cold rolling was 454.34 MPa. As the annealing temperature increased, the UTS gradually decreased due to crystal recovery, whereas the EL increased. When the sample started to recrystallize, the UTS dropped sharply and was reduced to 342.73 MPa after complete recrystallization, and it remained stable in the range of 360–400 °C. After that, as the temperature increased, the recrystallized grain continued to increase and the UTS and EL gradually reduced. For the 5182-0.4Ce Al alloy, the recrystallization started at about 340 °C and finished at about 360 °C, which was consistent with the 5182-0Ce Al alloy. The UTS of the 5182-0Ce Al alloy after cold rolling was 414.65 MPa, which was reduced to 322.16 MPa after complete recrystallization. With the increase of the annealing temperature after recrystallization, the UTS of the 5182-0Ce Al alloy continuously decreased to 314.54 MPa after annealed at 400 °C.

### 3.2. Microstructure Evolution

**Figure 3:**
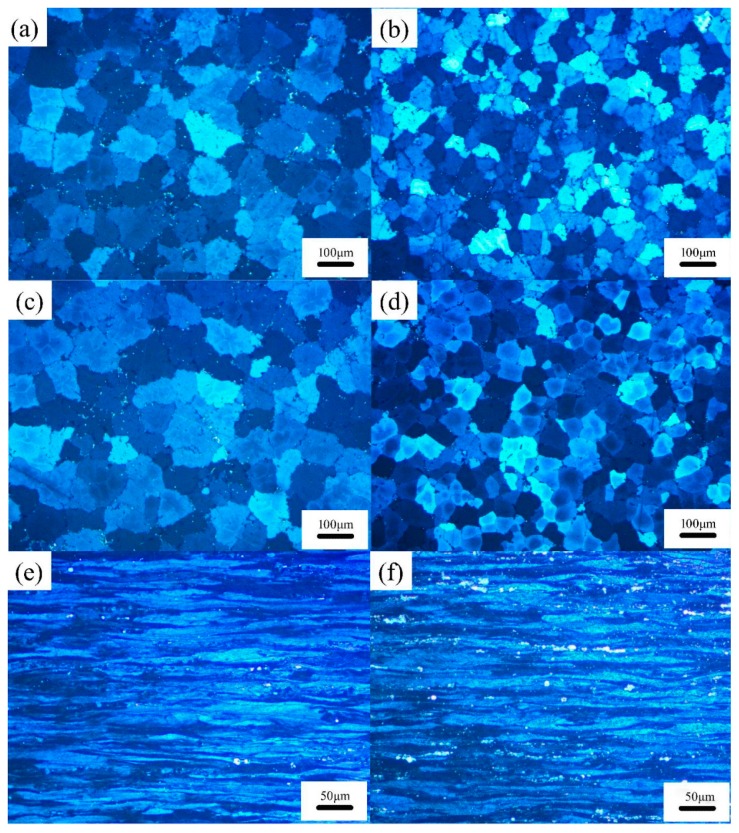
The microstructure of the 5182 Al alloys before and after Ce-modification. 5182-0Ce Al alloy: (**a**) as-cast, (**c**) homogenized annealed, and (**e**) cold-rolled. 5182-0.4Ce Al alloy: (**b**) as-cast, (**d**) homogenized annealing, and (**f**) cold rolled.

**Figure 4:**
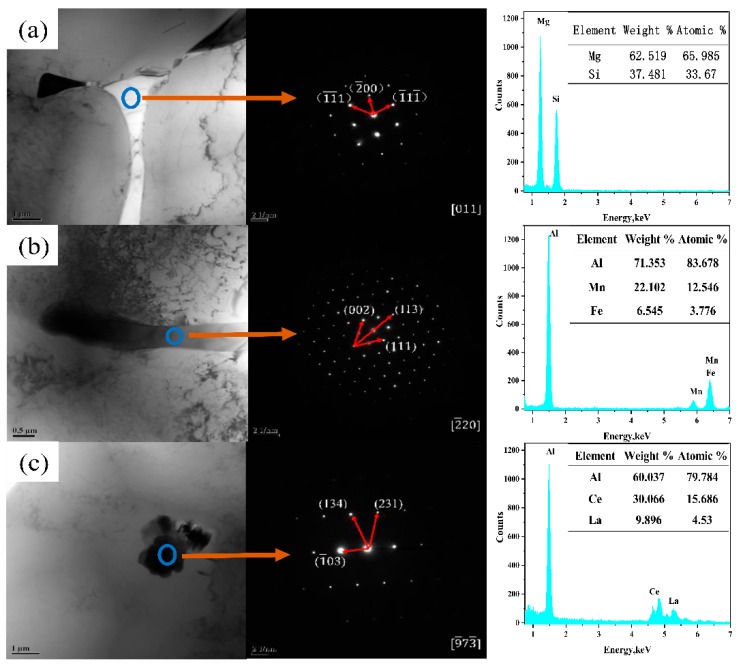
TEM images, Selected Area Electron Diffraction (SAED) and energy-dispersive X-ray diffraction (EDS) of the as-cast 5182 Al alloy: (**a**,**b**) 5182-0Ce and (**c**) 5182-0.4Ce.

**Figure 5:**
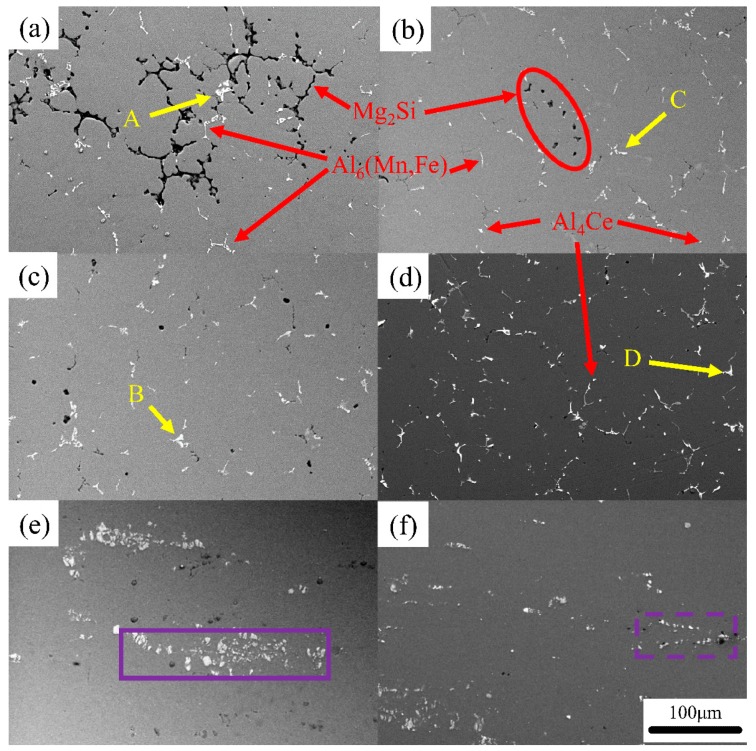
SEM images of the 5182 Al alloys before and after Ce-modification. 5182-0Ce Al alloy: (**a**) as-cast, (**c**) homogenized annealing, and (**e**) cold-rolled; 5182-0.4Ce Al alloy: (**b**) as-cast, (**d**) homogenized annealing, and (**f**) cold rolled.

The microstructures of the 5182 Al alloys before and after Ce-modification were compared (Figure 3). It was evident that the α-Al in both Al alloys showed an obvious dendritic structure, and the secondary phases were distributed along the grain boundaries. In comparison with the 5182-0Ce Al alloy, the number of dendrites in the 5182-0.4Ce Al alloy significantly increased, and the grains were refined (Figure 3a,b). Homogenized annealing had little effect on the grain size in the two types of alloys (Figure 4a,b). The grains of the alloys were elongated by the pressure on the roll along the rolling direction, and they appeared as slender fibrous shapes (Figure 4a and Figure 5a). It was noteworthy that the grain aspect ratio of the 5182-0.4Ce Al alloy was large. Additionally, it is worth noting that the concentration of precipitates in the 5182-0.4Ce Al alloy was quite different from that in the 5182-0Ce Al alloy.

The secondary phase in the 5182 Al alloy was confirmed by using TEM because the precipitate content in the alloy was lower than the X-ray diffraction (XRD) detectable accuracy. Two different secondary phases could be distinguished in the as-cast 5182-0Ce Al alloy (Figure 4a,b). The EDS result (Figure 4a) corresponding to the bright white secondary phase showed that the region consisted of Mg and Si, and its atomic ratio was approximately 2:1. It could be further confirmed as Mg_2_Si by SAED. The EDS result (Figure 4b) corresponding to the dark black secondary phase contained Al\Mn\Fe. It could be further confirmed as Al_6_(Fe,Mn) with a prototype of Al_6_Mn by SAED. In the 5182-0.4Ce Al alloy, the presence of the Al_4_(Ce,La) phase with a prototype of Al_4_Ce was confirmed by EDS and SAED (Figure 4c), which was consistent with previous research results [3,19,20].

The microstructure evolutions of Mg_2_Si, Al_6_(Mn, Fe), and Al_4_(Ce,La) in the as-cast, homogenized annealed, and cold-rolled 5182 Al alloys, before and after Ce-modification, were observed (as shown in Figure 5). The results of EDS analysis at points A, B, C, and D of Figure 5 are presented in Table 3.

In the case of the 5182-0Ce Al alloy, the Mg_2_Si phase was black and distributed in the grain boundaries (Figure 5a). The Mg_2_Si phase was partially dissolved during the homogenized annealing process (Figure 5c) and was discretely distributed after the subsequent cold rolling process (Figure 5e). The addition of Ce-rich mischmetal to the 5182 Al alloy caused the network structure of the Mg_2_Si phase in the as-cast state to be broken (as shown in Figure 5b as the region of the red ellipse). After homogenized annealing, most of the Mg_2_Si phase was dissolved (Figure 5d), making it difficult to find in the cold-rolled state (Figure 5f).

The Al_6_(Mn, Fe) phase appeared in bright white in the 5182-0Ce Al alloy distributed along grain boundaries (Figure 5a). The Al_6_(Mn, Fe) phase could not be re-dissolved by homogenized annealing (Figure 5c), and this phase was crushed into fine particles during the rolling process and streamlined distribution (Figure 5e). It is worth noting that some of Al_6_(Mn, Fe) particles rich in Cr and Si atoms (Figure 5a, point A) were found in the 5182-0Ce Al alloy. The Cr and Si atoms could not be entirely diffused from the Al_6_(Mn, Fe) phase into α-Al during the homogenized annealing (Figure 5b, point B). After cold rolling, these particles still maintained a large size and were mixed with fine particles (Figure 5e, in the region of the solid purple line).

**Figure 6:**
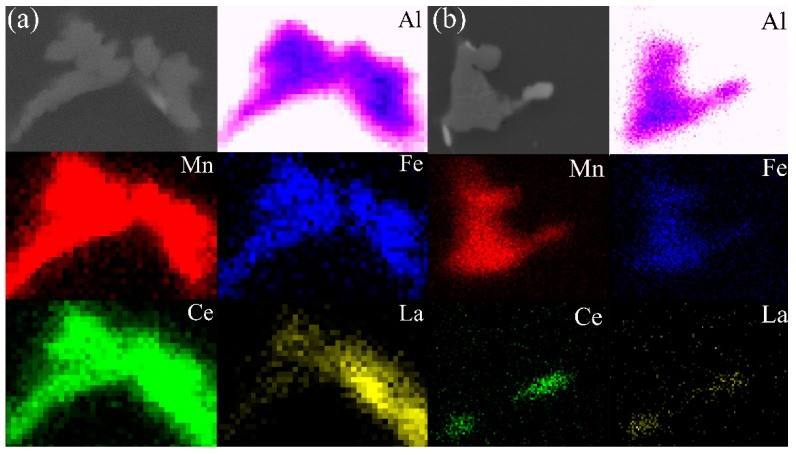
SEM images and EDS maps of the Ce-rich Al_6_(Mn,Fe) phase before (**a**) and after homogenized annealing (**b**).

In the 5182-0.4Ce Al alloy, the Al_6_(Mn, Fe) phase appeared gray–white in color and the bright white phase seemed to contain Ce due to the higher atomic number of Ce [3]. Ce existed in two primary forms. One was in the Al_4_(Ce,La) phase that was formed with Al (Figure 5b), and the other was solid-solved into the Al_6_(Mn, Fe) phase (Figure 5b, point C). Different from the case of the Al_6_(Mn, Fe) phase that contained Cr and Si, the Ce-rich Al_6_(Mn, Fe) phase was more significant in size after homogenized annealing (Figure 5b, point D), but it was crushed into fine particles during cold rolling (Figure 5f, the area of purple dash line). The Ce-rich Al_6_(Mn, Fe) phase before and after homogenized annealing was observed by EDS maps (Figure 6) to analyze the behavior of the Ce-rich Al_6_(Mn, Fe) phase that was rolled into fine particles. It was evident that, in contrast to the as-cast state, Ce particles diffused out from the Ce-rich Al_6_(Mn, Fe) phase and formed the Al_4_(Ce,La) phase at the edge of the Al_6_(Mn, Fe) after homogenized annealing.

**Figure 7:**
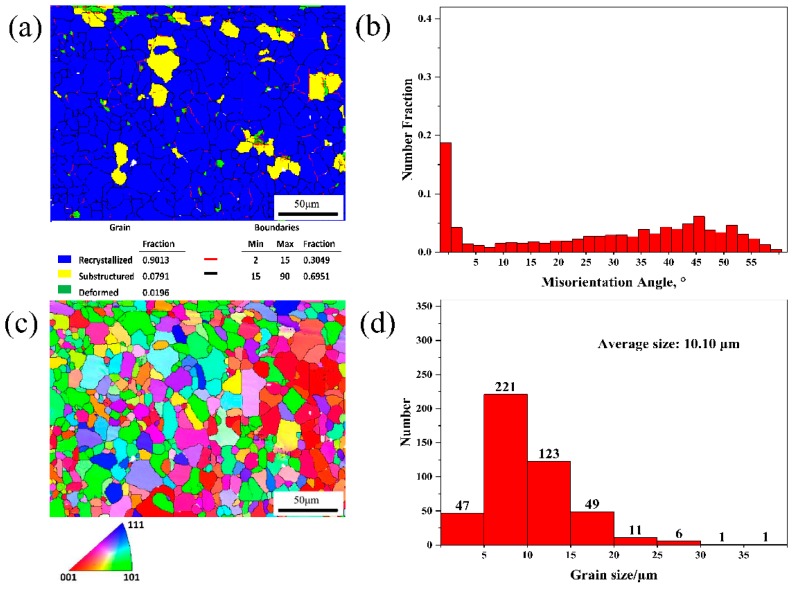
EBSD analyses of the 5182-0Ce Al alloy, which was cold rolled and then annealing at 360 °C. (**a**) Recrystallization grain distribution, (**b**) misorientation angles distribution, (**c**) IPF, and (**d**) grain size distribution.

**Figure 8:**
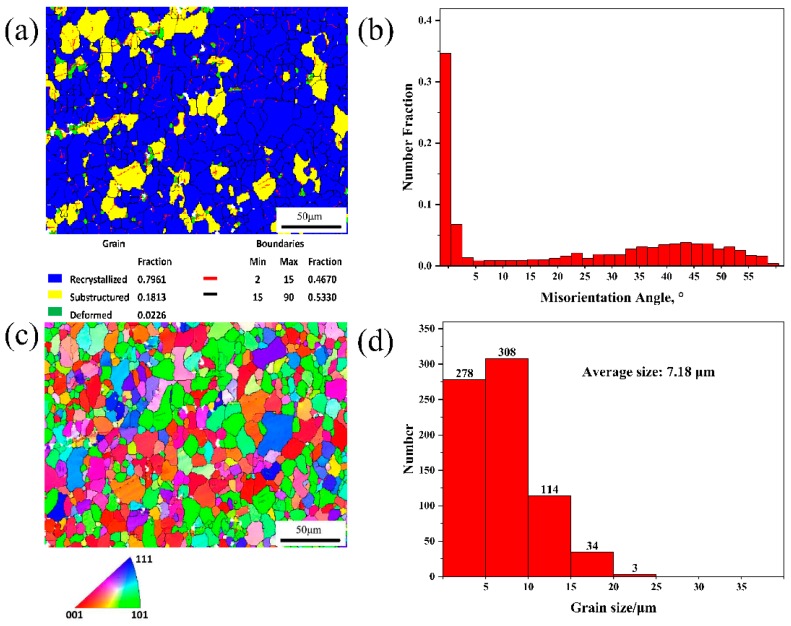
EBSD analyses of the 5182-0.4Ce Al alloy, which was cold rolled and then annealing at 360 °C. (**a**) Recrystallization grain distribution, (**b**) misorientation angles distribution, (**c**) IPF, and (**d**) grain size distribution.

To analyze the recrystallization behavior of the two alloys, EBSD analyses of the 5182-0Ce and 5182-0.4Ce Al alloys during cold rolling and annealing at 360 °C are shown in Figure 7 and Figure 8, respectively, from which we can analyze the recrystallization behavior of the two alloys. Based on analysis and calculations done using Channel 5 software, it can be seen that the fractions of subgrains in the 5182 Al alloy before and after Ce-modification were 7.91% and 18.13%, respectively (Figure 7a and Figure 8a). As can be observed in the low angle grain boundaries (LAGBs) in the subgrains, there were more LAGBs in the subgrains of the 5182-0.4Ce Al alloy, which was consistent with the distribution of the misorientation angles, in which the fraction of LAGBs in the 5182-0Ce Al alloy accounted for 30.49% of the angles, whereas this fraction in the 5182-0.4Ce Al alloy accounted for 46.7% (Figure 7a,b and Figure 8a,b). Figure 7c,d shows the Invers pole figure (IPF) and grain size distribution of the 5182-0Ce Al alloy, respectively. Grains smaller than 10 μm accounted for 58.39% of the grains, which had an average size of 10.10 μm. Figure 8c,d shows the IPF and grain size distribution of the 5182-0.4Ce Al alloy, respectively. Grains smaller than 10 μm in the 5182-0.4Ce Al alloy accounted for 79.51% of the grains, which had an average size of 7.18 μm.

## 4. Discussions

### 4.1. Effect of Ce-Rich Mischmetal on Microstructure Evolution

The mismatch between the Ce-rich mischmetal (Ce: 0.182 nm; La: 0.187 nm) and the Al atom (0.143 nm) was about 27.3% [16]. Therefore, it was difficult for Ce atoms to dissolve in the α-Al lattice or to substitute Al atoms in the lattice with Ce atoms. With the nucleation and growth of α-Al dendrites, a large amount of Ce atoms were enriched at the frontiers of the solid–liquid interface, and this enrichment led to local compositional undercooling [20], which helped stimulate the flourishing of the α-Al dendrites and therefore resulted in a grain refinement effect (Figure 3a,b). The finer grains in the as-cast 5182-0.4Ce Al alloy evolved into a larger grain aspect ratio than the unmodified sample after the same processing (Figure 3e,f).

The enrichment of Ce elements at the frontiers of the solid–liquid interface also decreased the diffusion rate of Mg and Si atoms from α-Al lattice to the liquid phase, thereby reducing the efficiency of solute redistribution [21]. Thus, the nucleation and growth of Mg_2_Si in the 5182 Al alloy was retarded, which caused the breakage of the Mg_2_Si network in the 5182-0.4Ce Al alloy (Figure 5a,b). Nevertheless, since the Mg_2_Si phase was re-dissolved during homogenized annealing, its influence on the microstructure evolution of both of alloys had almost no difference.

The remaining Ce elements wrapped around the primary Al_6_(Mn, Fe) phase (precipitated at 658 °C [3]), thus preventing Cr and Si atoms from diffusing into the primary Al_6_(Mn, Fe) phase. Due to the fact that the Ce elements almost dissolved in the α-Al lattice, the Cr and Si atoms diffused into the grain interior during homogenized annealing, while the Ce elements diffused at grain boundaries. Following the principles of the Zener–Hillert diffusion mechanism, it is evident that the diffusion rate of atoms at the grain boundary was much higher than that in the grain interior; in fact, the diffusion rate of Si atoms in the grain boundary was 1000 times than that in the grain interior [22]. Therefore, the Al_6_(Mn, Fe) phase rich in Cr and Si atoms could not be effectively dissolved (Figure 5c), and the Ce-rich Al_6_(Mn, Fe) phase formed the Al_4_(Ce,La) phase at the edge (Figure 6), which reduced the distortion energy of the Al_6_(Mn, Fe) phase and made it easier to be crushed into fine particles during the cold rolling process (Figure 5e,f). In summary, the addition of the Ce-Rich Mischmetal made the Al_6_(Mn, Fe) phase finer after cold rolling.

The nucleation of recrystallized grains was related to the dislocation motion in the subgrain and the movement of LAGBs between adjacent subgrains [23]. The secondary phase particles could have played a pinning effect on grain boundary and dislocation motion [24]. The finer the secondary phase particles, the greater the hindrance [15]. Therefore, the recrystallized grain size was refined from 10.01 to 7.18 μm by Ce-modification (Figure 7c and Figure 8c). Additionally, the large-sized secondary phase particles in the 5182-0Ce Al alloy promoted the preferential nucleation of the partially recrystallized grains [25]. These grains were large after recrystallization, resulting in unevenness in grain sizes (Figure 7d and Figure 8d).

### 4.2. Effect of Ce-Rich Mischmetal on Mechanical Properties

The addition of the Ce-rich mischmetal increased the UTS of the 5182 aluminum alloy after cold rolling from 414.65 to 454.34 MPa, and the UTS after recrystallization increased from 322.16 to 342.73 MPa. The strengthening mechanisms of the cold-rolled 5182 Al alloy and the recrystallized annealed 5182 Al alloy were investigated.

The microstructure modification of the Ce-rich mischmetal on the cold-rolled 5182 Al alloy was mainly reflected in a larger grain aspect ratio and finer Al_6_(Mn, Fe) particles (Figure 3e,f and Figure 5e,f) after deformation. It was reasonable to consider that the increment of the strength of the cold-rolled 5182 Al alloy could be determined by cell-size strengthening, dislocation density strengthening, and particle strengthening [26], as the solid solution strengthening could be ignored because not only the Mg_2_Si phase was re-dissolved during the homogenized annealing (Figure 5b,c) but also two alloys have a similar solute element content (Table 2). The increment of the strength of the recrystallization annealed 5182 Al alloy could be calculated by using the Hall–Petch equation [8,27]:

(1) $$\Delta \sigma =K\left(D_{1}{}^{-1/2}-D_{2}{}^{-1/2}\right)$$

where K (=0.040MPam^−1/2^ [28]) was an experimental constant and $D_{1}$ and $D_{2}$ were the recrystallized grain sizes of the 5182 Al alloy before and after Ce-modification. Therefore, the calculated Δσ was approximately 19.9 MPa, which was consistent with the present experimentally obtained data. After recrystallization annealing, the UTS of the 5182-0Ce Al alloy continuously decreased with the increase of annealing temperature, which was related to the recrystallization grain unevenness [29].

## 5. Conclusions

- The addition of Ce-rich mischmetal refined the as-cast *α*-Al grains, making the grain aspect ratio larger after cold rolling. Moreover, the recrystallized grains were refined from 10.01 to 7.18 μm.
- Ce-rich mischmetal addition prevented Cr and Si atoms of the solid-solve from diffusing into the Al_6_(Mn, Fe) phase, which made it refine after cold rolling. In the 5182-0.4Ce Al alloy, the network structure of the Mg_2_Si phase in the as-cast state was broken.
- Ce-rich mischmetal addition enhanced the mechanical properties of the 5182 Al alloy. The UTS of the cold-rolled 5182 Al alloy increased by 9.6% through cell-size strengthening, dislocation density strengthening, and particle strengthening. The UTS of the recrystallization annealed 5182 Al alloy increased by 6.5% through grain size strengthening. After recrystallization annealing, the UTS of the 5182-0.4Ce Al alloy was more stable than that of the 5182-0Ce alloy.

## Figures and Tables

**Table 1:** Chemical composition of the Ce-rich mischmetal (wt%) [17].

Element	Ce	La	Pr	Nd	Sm	Al
Content	6.95	1.54	0.32	0.58	0.10	Bal

**Table 2:** Chemical composition of the 5182 aluminum alloy, (wt%).

	Re	Mg	Mn	Fe	Si	Cr	Ti	Cu	Zn	Al
5182-0Ce	-	4.90	0.35	0.34	0.17	0.05	0.11	0.06	0.13	Bal
5182-0.4Ce	0.36	4.93	0.36	0.31	0.16	0.08	0.06	0.06	0.14	Bal

**Table 3:** SEM-EDS analysis results corresponding to Figure 5.

	Al	Mg	Mn	Fe	Si	Cr	Ce	La	Total
**A**	69.30	4.21	16.33	5.09	1.85	3.21	-	-	100
**B**	70.06	4.18	17.20	4.71	1.82	2.03	-	-	100
**C**	68.06	2.44	17.10	3.58	-	-	6.55	2.27	100
**D**	71.82	3.63	15.82	4.34	-	-	2.96	1.43	100
